# Conditional Piety

**Published:** 1910-06

**Authors:** 


### Conditional Piety.

Two Scotch fishermen, James and Sandy, belated and befogged on a rough water, were in some trepidation lest they should never get ashore again. At last Jamie said:

"Sandy, I'm steering, and I think you'd better put up a bit of prayer."

"I don't know how," said Sandy.

"If ye don't I'll chuck ye overboard," said Jamie.

Sandy began: "Oh, Lord, I never asked anything of Ye for fifteen years, and if Ye'll only get us safe back, I'll never trouble Ye again, and—"

"Whist, Sandy," said Jamie. "The boat's touched shore; don't be beholden to anybody."—*Short Stories.*

